# Evaluation of the Efficacy of Excimer Laser Ablation of Cross-Linked Porcine Cornea

**DOI:** 10.1371/journal.pone.0046232

**Published:** 2012-10-03

**Authors:** Shihao Chen, Yini Li, Aleksander Stojanovic, Jia Zhang, Yibo Wang, Qinmei Wang, Theo Seiler

**Affiliations:** 1 The Affiliated Eye Hospital of Wenzhou Medical College, Lucheng District, Wenzhou, Zhejiang, People's Republic of China; 2 Eye Department, University Hospital of North Norway and Synslaser Kirurgi AS, Tromsø, Norway; 3 Institute for Refractive and Ophthalmic Surgery, Zurich, Switzerland; University of Reading, United Kingdom

## Abstract

**Background:**

Combination of riboflavin/UVA cross-linking (CXL) and excimer laser ablation is a promising therapy for treating corneal ectasia. The cornea is strengthened by cross-linking, while the irregular astigmatism is reduced by laser ablation. This study aims to compare the efficacy of excimer laser ablation on porcine corneas with and without cross-linking.

**Methods and Findings:**

The porcine cornea was de-epithelialized and treated with 0.1% riboflavin solution for 30 minutes. A half of the cornea was exposed to UVA-radiation for another 30 minutes while the controlled half of the cornea was protected from the UVA using a metal shield. Photo therapeutic keratectomy (PTK) was then performed on the central cornea. Corneal thickness of 5 paired locations on the horizontal line, ±0.5, ±1.0, ±1.5, ±2.0, and ±2.5 mm from the central spot, were measured using optical coherence tomography prior to and after PTK. The ablation depth was then determined by the corneal thickness. There was a 9% difference (P<0.001) in the overall ablation depth between the CXL-half corneas (158±22 µm) and the control-half corneas (174±26 µm). The ablation depths of all 5 correspondent locations on the CXL-half were significantly smaller (P<0.001).

**Conclusion:**

The efficacy of the laser ablation seems to be lower in cross-linked cornea. Current ablation algorithms may need to be modified for cross-linked corneas.

## Introduction

Keratoconus is a bilateral, non-symmetrical, progressive corneal degenerative disorder. There are many approaches available to correct the refractive errors caused by keratoconus, such as spectacles, rigid gas-permeable contact lenses, intracorneal ring segments, and photorefractive keratectomy. However, none of the treatments above have shown successful control on the progression, and it leaded to corneal transplantation in progressive cases. Seilor first introduced corneal collagen cross-linking (CXL) which is a method to biomechanically stabilize the cornea and stop the progression of keratoconus [1]. This method has raised a new hope in treating not only keratoconus [2] and pellucid marginal degeneration [3], but also laser-assisted in situ keratomileusis-induced keratectasia [4], radial keratotomy-induced keratectasia [5], [6], keratitis [7], [8], and bullous keratopathy [9], [10]. Even though CXL suppresses the progression, the vision does not improve much because of the remained refractive error [2], [11], [12]. Therefore, the combination with photorefractive keratectomy (PRK) or phototherapeutic keratectomy (PTK) was introduced for optical regularization [13], [14]. There are currently two modes of the combination surgery, one using laser treatment right before CXL [14], [15], [16], and the other, based on laser treatment 6–12 months after CXL [13]. The aim of the current study is to assess excimer laser ablation efficacy in CXL treated cornea.

## Methods

### Preparation

Thirty porcine eyes from the local abattoir (Meat Processing Plant of Wenzhou Shopping Basket Group Co., Ltd., Wenzhou, Zhejiang, China) were conserved in containers at 4°C immediately after enucleation and were used within 2 to 10 hours post-mortem. To minimize the error caused by stromal hydration, only clear corneas with intact epithelium and no initial edema were selected. To avoid potential error due to individual variations, one half of each cornea was cross-linked and the other half served as a control. Nasal-temporal comparisons were preferred to superior-inferior ones since porcine corneal thickness (CT) differs least along this line [17]. All experiments were performed at 22°C and 60% humidity.

**Figure 1:**
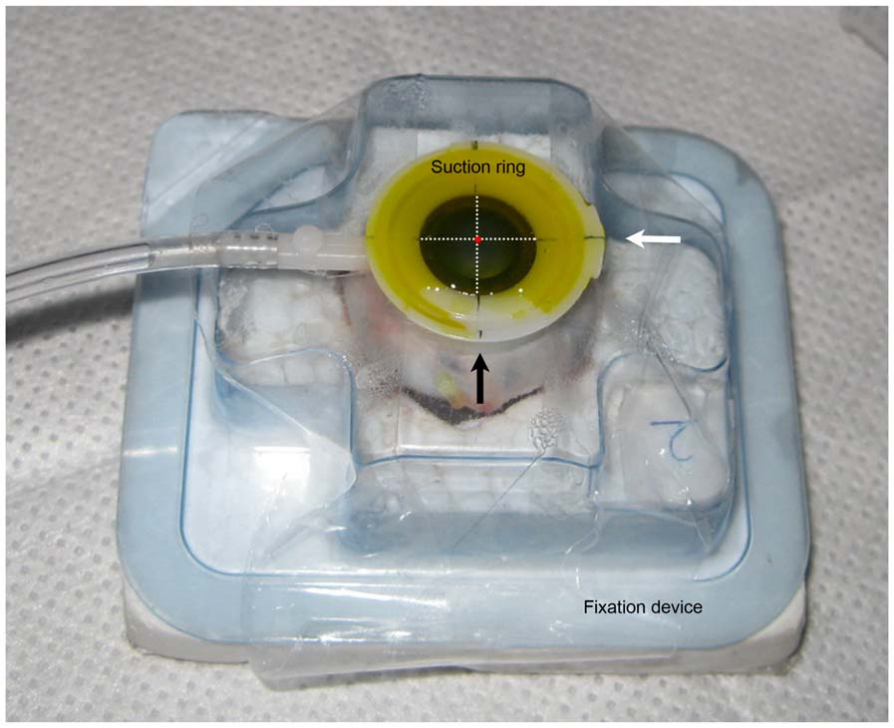
Porcine eye was mounted in a fixation device throughout the experiment. Each eye was mounted in a fixation device throughout the experiment. A 9.0 mm suction ring with a cross-mark was applied to the eye and centered on the corneal apex to avoid eye rotation. The vertical mark (black arrow) on the suction ring separated the cornea into CXL-half and control-half, while the horizontal line (white arrow) on the suction ring represented the direction along which the CT was measured. The intersection (red spot) of the two lines (dotted lines, invisible) was used as the center spot of pachymetry and laser ablation. In this picture, riboflavin solution (yellow colored) filled within the suction ring and formed a thin and intact film over the de-epithelialized cornea throughout the CXL procedure.

Physiological saline solution was injected into the vitreous to maintain a stable and optimized intraocular pressure (Tn), which was estimated by digital palpation that estimated intraocular pressure by gently pressing the index finger against the cornea in this study. Each eye was then mounted in a fixation device with a 9.0 mm suction ring centered on the apex throughout the experiment to avoid eye rotation (Figure 1). The vertical line on the suction ring separated the cornea into a CXL-half and control-half, while the horizontal line on the suction ring represented the direction along which the CT was measured. The intersection of the two lines was used as the central spot of pachymetry and laser ablation. To avoid any influence of light, all the eyes were stored in a dark room throughout the experiment.

### Corneal Collagen Cross-linking (CXL)

**Figure 2:**
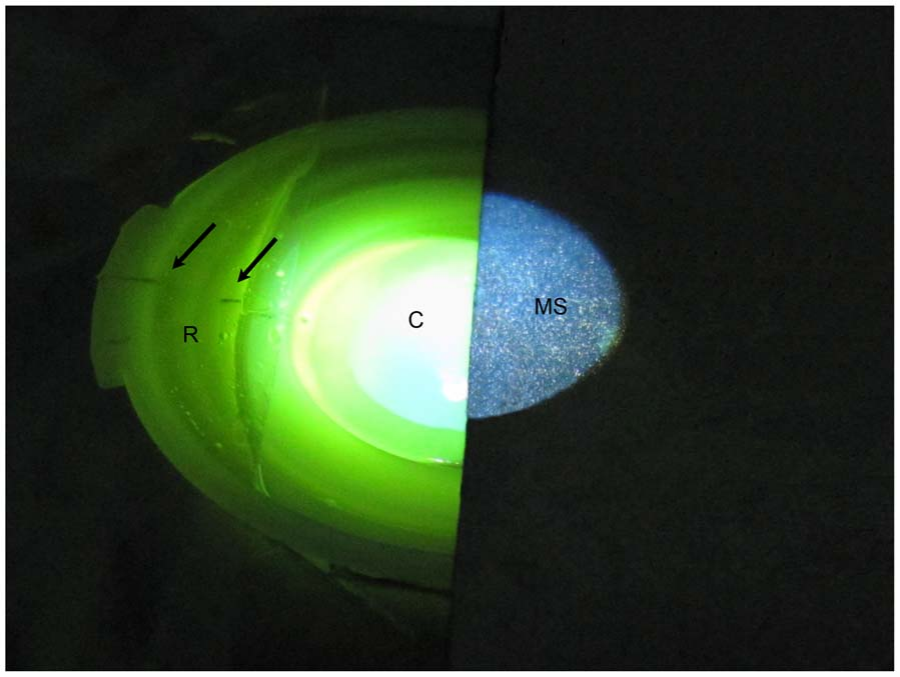
Porcine eye underwent UVA-irradiation with half of the cornea sheltered by a metal shield. During the irradiation, one half of the cornea was sheltered with a metal shield (MS) (without contact) so that only the uncovered half (C) was irradiated and cross-linked. The edge of the shield was in accordance with the vertical line on the suction ring (R). Only part of the horizontal line (black arrow) could be seen.

After de-epithelialization of 9 mm central cornea, 0.1% riboflavin solution (0.1% riboflavin, 20% dextran-T-500, MEDIO-CROSS 3.0 mL isotonic solution, Kiel, Germany) was applied for 30 minutes. Riboflavin filled within the suction ring and formed a thin and intact film throughout the CXL procedure (Figure 1). Prior to irradiation, one half of the cornea was sheltered with a metal shield (without contact) so that only the uncovered half was irradiated (Figure 2) as only the irradiated cornea could be cross-linked [18]. The desired irradiance of a UV lamp was calibrated using a UVA meter. The exposed half of the cornea was then irradiated by UVA for another 30 minutes with an irradiance of 3 mW/cm^2^ (dose 5.4 J/cm^2^) 5 cm away from the cornea using the 370 nm UV-lamp (UV-X, IROC AG, Zurich, Switzerland). Riboflavin film was wiped off right before pachymetry.

### Pachymetry

**Figure 3:**
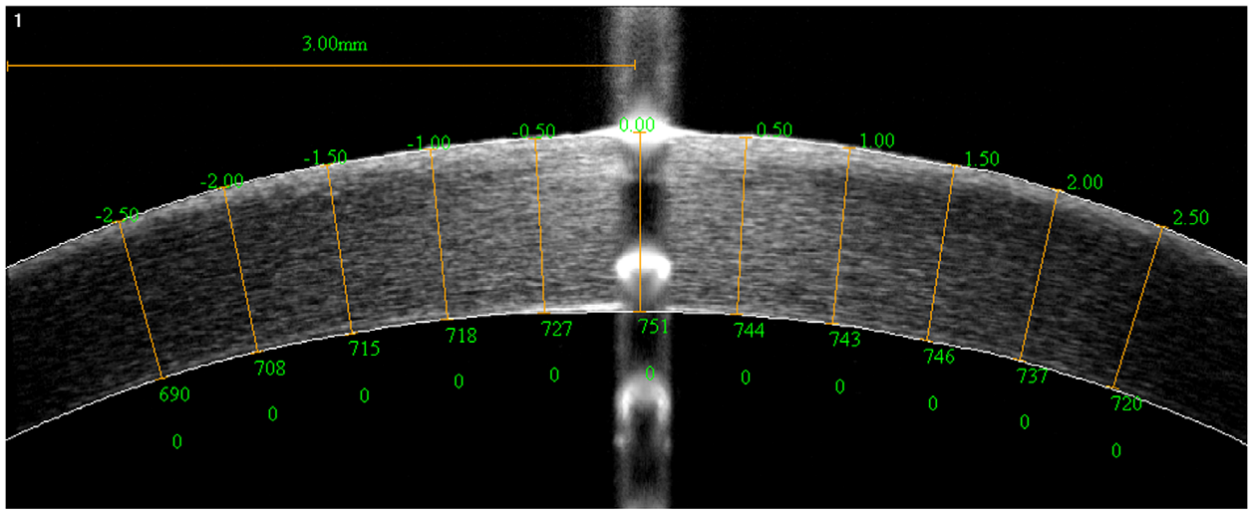
Pachymetry by OCT. Pachymetry was taken along the horizontal line of the suction ring using OCT. CT was measured on that meridian at 5 pairs of equidistant locations, ±0.5, ±1.0, ±1.5, ±2.0, and ±2.5 mm from the central spot, using the RTVue's “flap tool” in the CL-line scan.

Pachymetry was performed prior to and after PTK using optical coherence tomography (OCT) (RTVue-100, Optovue, Inc., Fremont, CA, USA). The horizontal line and the central spot on the suction ring were placed on the OCT's horizontal meridian and measurement center, respectively. Six OCT images were captured consecutively on each eye and the three best images, with regard to centration in both vertical and horizontal direction, as well as to absence of tilt or rotation, were selected for analysis. CT was measured at 5 pairs of equidistant locations at ±0.5, ±1.0, ±1.5, ±2.0, and ±2.5 mm away from the central spot, using the RTVue's “flap tool” in the CL-line scan (Figure 3). To minimize the influence of dehydration in the stroma, the time interval of the measurements was standardized.

### Phototherapeutic Keratectomy (PTK)

The excimer laser (WaveLight Laser Technology, AG, Erlangen, Germany) ablation (400 Hz) was centered on the central spot by projecting the laser's red cross on the cross-mark of the suction ring. A circular PTK ablation of 6.0 mm in diameter and 50 µm in depth was repeated 3 times to reach the intended ablation depth of 150 µm. By this method both CXL- and control-halves were ablated simultaneously.

### Statistical Analysis

Laser ablation depth was calculated by subtracting the post-PTK CT from the pre-PTK CT and was compared using paired T-test. Multiple linear regression analysis was used to analyze the association between ablation depth and distance from the central spot. The statistical analysis was performed with the statistical package for social sciences (SPSS 17.0 GmbH, Munich, Germany). Statistical significance was defined as p<0.05.

## Results

**Figure 4:**
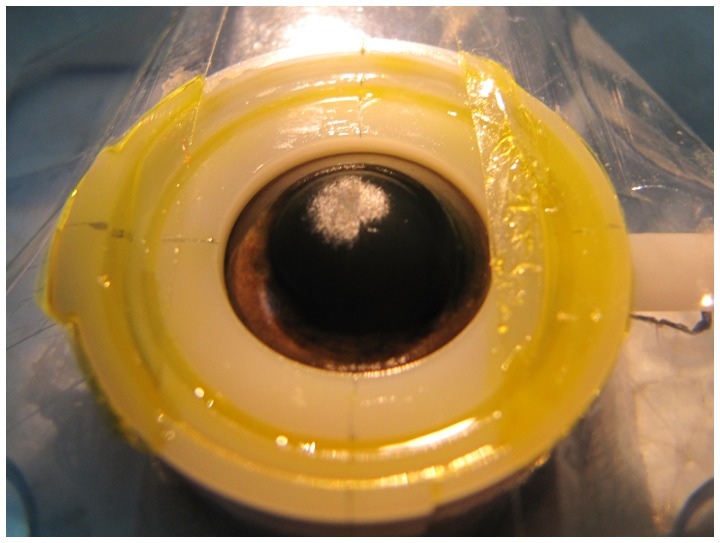
Demarcation line observed after laser ablation. A distinct demarcation line was observed between CXL-half (left) and control-half (right) after excimer laser ablation, with the former showing lower reflectivity.

In all 30 eyes, the CXL-halves appeared less reflective comparing with control-halves after CXL and this property became more obvious after PTK (Figure 4).

**Figure 5:**
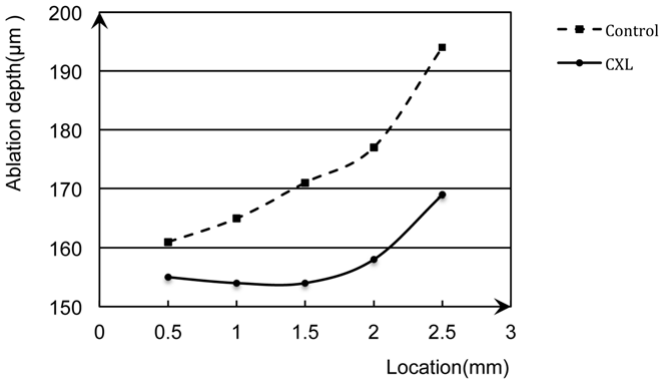
Mean ablation depth of 30 eyes at the five correspondent locations. The horizontal axis represented the measured location of the cornea, the vertical axis represented the ablation depth. The solid line represented the CXL group that was less ablated. The dotted line represented the control group that was more ablated. The peripheral locations appeared to be more ablated than central locations.

The average ablation depths at the 5 correspondent locations were shown in Table 1. There was a 9% difference (p<0.001) in the overall ablation depth between the CXL-half corneas (158±22 µm) and the control-half corneas (174±26 µm). Moreover, the ablation depths were significantly smaller on the CXL-halves comparing with the control-halves at all 5 correspondent locations (Figure 5).

**Table 1:** Average ablation depths in the 5 paired locations.

Location*(mm)	Ablation depth (µm)		P‡
	CXL†	Control†	
0.50	155±21	161±23	<0.001
1.00	154±21	165±24	<0.001
1.50	154±22	171±24	<0.001
2.00	158±21	177±23	<0.001
2.50	169±22	194±23	<0.001
Overall	158±22	174±26	<0.001

* : The distance from the central spot; †: Average of 30 eyes per group ± standard deviation. CXL represented the CXL group. Control represented the control group. Overall represented the average ablation depth of all the measured locations in 30 eyes per side; ‡: P-value from paired T-test.

The ablation depths were strongly correlated with both the CXL group (R = 0.999, p = 0.038) and control group (R = 0.985, p = 0.015). In each group, the laser ablation depths significantly increased as a function of distance from the central spot, with the peripheral locations being more ablated than the central ones. As the central ablation depth set to be 150 µm, the best-fit functions were displayed as follow:

The CXL-half: Y = 151.414+12.558X-14.697X^2^+5X^3^

The control-half: Y = 163.751−7.590X+7.752X^2^

Y represents the laser ablation depth (µm) and X represents the distance (mm) from the central spot.

## Discussion

Ever since its clinical introduction by Schnitzler in 2000 [19], riboflavin/UVA-induced CXL has proven to be a promising treatment for keratoconus. This photochemical reaction increases new intrafibrillar bonds in corneal stroma [2], thus changes corneal properties, including increasing collagen fiber diameter [20] and enhancing biomechanical rigidity [21]. There is similar distribution of riboflavin [22], [23] and stiffening effect [22] in porcine and human corneas, and the maximal effect of CXL is limited to the anterior 300 µm [21]. When the PTK ablation is intended to the anterior 150 µm of porcine corneas in our research, the data obtained are of reference value. Still, further research is needed before it can be clinically applicable to human eyes.

Corneal stromal demarcation lines at a depth of 300 µm were observed two weeks after CXL, and it is presumed that the lines may be due to the differences in refractive index and/or reflection properties between corneal layers with and without CXL [24]. In accordance with these findings, we observed an apparent difference in surface reflectivity between the CXL- and control-halves of the cornea.

Kanellopoulos [13] has demonstrated the effects of CXL on corneal ablation on a patient with keratoconus. It was stated that a more rigid cornea might have an ablation rate different from a normal cornea, so the one-year-old CXL treated eye was 25% undercorrected using PRK to obtain the optimized effect. Therefor, we investigated the ablation rate using porcine corneas in this study and found a significant smaller ablation depth in the CXL-half corneas compared to that in the control-half.

The interaction of 193 nm excimer laser with the corneal tissue represents a photochemical effect, each photon supplies enough energy to directly break a molecular bond and each pulse effectively removes certain amount of corneal tissue [25], [26]. If equal energy is required in breaking CXL-induced bonds and original bonds, we may speculate that breaking the increased bonds in the CXL-half demands extra energy and totally more pulses than the control-half. As the same amount of laser energy was applied to both sides of cornea in this study, the CXL strengthened half was less ablated. The conventional ablation rate of 0.23 to 0.30 µm/mm^2^/pulse [27], [28], [29] should be adjusted as 0.21 to 0.27 µm/mm^2^/pulse accordingly.

In this study, the ablation depth increased from center towards periphery on both the CXL- and control-halves. This is probably due to the PTK ablation profile and the different curvature of porcine cornea compared to that of human one. The current PTK ablation profile designed for human cornea produces more pulses in mid-periphery in order to compensate for energy loss in mid-periphery. This peripheral compensation is excessive for porcine cornea due to its flatter curvature compared to human cornea [30]. As a result, the periphery is more ablated than the center.

Amount of corneal hydration between CXL and control should be identical. The pre-ocular isotonic riboflavin film played an important role in preventing the de-epithelialized and exposed cornea from dehydration [31], [32]. In addition, the slight decrease in CT has nothing to do with UVA irradiation [33]. Unlike Kampik's research in which different eyes were selected as CXL group and control group [34], each eye in our study was divided into CXL-half and control-half, so that both halves received the same amount of riboflavin solution and maintained identical hydration. Therefore, it is unlikely to induce the difference in CT between CXL- and control-half in our study.

A combination of CXL and excimer laser ablation is becoming a popular clinical treatment for corneal ectasia. In this study, the mean ablation depth in CXL porcine cornea was reduced by 9%, which may help to modify the ablation algorithms of excimer treatment for CXL treated eyes. However, our results reflected experimental study on porcine eyes, where the time interval between CXL and laser ablation is much shorter than that in a clinical situation, where the ablation may occur several months after CXL. More data based on human tissue is necessary.
